# Subretinal Implantation of Electrospun, Short Nanowire, and Smooth Poly(**ε**-caprolactone) Scaffolds to the Subretinal Space of Porcine Eyes

**DOI:** 10.1155/2012/454295

**Published:** 2012-03-15

**Authors:** A. T. Christiansen, S. L. Tao, M. Smith, G. E. Wnek, J. U. Prause, M. J. Young, H. Klassen, H. J. Kaplan, M. la Cour, J. F. Kiilgaard

**Affiliations:** ^1^Department of Ophthalmology, Glostrup Hospital, Copenhagen University Hospital, 2600 Glostrup, Denmark; ^2^Advanced Development Center, CooperVision, Inc., Pleasanton, CA 94588, USA; ^3^NuVention Solutions Inc., Valley View, OH 44125, USA; ^4^Case Western Reserve University, Cleveland, OH 44106, USA; ^5^Eye Pathology Institute, University of Copenhagen, 2100 Copenhagen, Denmark; ^6^Department of Ophthalmology, Schepens Eye Research Institute, Harvard Medical School, Boston, MA 02114, USA; ^7^The Gavin Herbert Eye Institute and Stem Cell Research Center, University of California, Irvine, CA 92697, USA; ^8^Department of Ophthalmology and Visual Sciences, Kentucky Lions Eye Center, University of Louisville, Louisville, KY 40208, USA

## Abstract

Biodegradable scaffolds play an important adjunct role in transplantation of retinal progenitor cells (RPCs) to the subretinal space. Poly(*ε*-Caprolactone) (PCL) scaffolds with different modifications were subretinally implanted in 28 porcine eyes and evaluated by multifocal electroretinography (mfERG) and histology after 6 weeks of observation. PCL Short Nanowire, PCL Electrospun, and PCL Smooth scaffolds were well tolerated in the subretinal space in pigs and caused no inflammation and limited tissue disruption. PCL Short Nanowire had an average rate of preserved overlying outer retina 17% higher than PCL Electrospun and 25% higher than PCL Smooth. Furthermore, PCL Short Nanowire was found to have the most suitable degree of stiffness for surgical delivery to the subretinal space. The membrane-induced photoreceptor damage could be shown on mfERG, but the reductions in P1 amplitude were only significant for the PCL Smooth. We conclude that of the tested scaffolds, PCL Short Nanowire is the best candidate for subretinal implantation.

## 1. Introduction

Subretinally transplanted retinal progenitor cells (RPCs) have in a number of animal models shown the ability to migrate to the outer retina, differentiate to mature photoreceptors, and generate synapses with existing cells [1]. As the mammalian retina does not regenerate [2], this provides a great restorative potential for retinal degenerative diseases. When transplanting cells to the subretinal space, the use of scaffolds has been shown to increase the number of delivered and surviving cells, to enable a more precise and localized delivery [3], and to promote differentiation and organization of grafted RPCs [3–5]. Furthermore, scaffolds can be loaded with regulatory and modulating drugs to further assist differentiation, function, and survival [6–8].

Several materials have been tested as scaffolds for RPC transplantation and found to support adhesion, survival, and migration [3, 9]. One of the more promising scaffold materials is poly(*ε*-caprolactone) (PCL) as it is mechanically compliant, nontoxic, degrades by slow surface erosion and can be fabricated as very thin membranes [9–11]. Scaffolds of PCL can be fabricated by different methods to produce specific structural and mechanical properties [9, 10, 12, 13]. Such types of PCL membranes are the electrospun (PCL-E), the Short Nanowire (PCL-SNW), and the Smooth (PCL-S). All three have previously been tested and for the PCL-E and PCL-SNW shown promising abilities for supporting growth, differentiation and migration of RPCs in vitro and in the case of PCL-SNW also in an in vivo study on mice [9, 13]

In this study, we transplanted naked PCL membranes to the subretinal space of pigs to obtain a baseline for the histological and electrophysiological effect of subretinal transplantation as well as long time presence of PCL-E, PCL-SNW, and PCL-S membranes in the eye. The size of the porcine eye enabled the use of surgical techniques and equipment used in the clinic, and thereby this study also evaluates the mechanical properties of the tested membranes in relation to the procedure of subretinal transplantation in humans. Voss Kyhn et al. [14] have shown the surgical steps of gaining access to the subretinal space to have no detectable effect on the multifocal electroretinogram (mfERG) in pigs 6 weeks post-surgically, and we assume that the electrophysiological effects seen arise only from the transplanted membranes. Also the immediate histological shortening of photoreceptor outer segments following bleb formation has been shown to normalized 6 weeks post-surgically [14]. We thereby intended this study to serve as histological and electrophysiological baseline for future RPC-PCL composite studies.

## 2. Material and Method

All experiments were performed in compliance with the ARVO Statement for the Use of Animals in Ophthalmic and Vision Research. The Danish Animal Experiments Inspectorate granted permission for the use of the animals (permission 2007/561-768). Trained veterinary nurses and technicians carried out all handling of the animals.

A total of 28 female domestic pigs of Danish Landrace/Duroc/Hampshire/Yorkshire breed were used for these experiments (age 3-4 months; weight 23–30 kg). Only left eyes underwent membrane implantation. The animals were premedicated with Tiletamine 1.19 mg/kg, Zolazepam 1.19 mg/kg (Zoletil 50 Vet Virbac SA, Carros, France), Methadone 0.24 mg/kg (Nycomed, Roskilde, Denmark), Ketamine 1.43 mg/kg (Intervet, Skovlunde, Denmark), and Xylazine 1.24 mg/kg (Intervet, Skovlunde, Denmark). Thereafter, anesthesia was maintained with continuous intravenous infusion (i.v.) of propofol 15 mg/kg/h (Fresenius Kabi, Bad Homburg, Germany). The animals were endotracheally intubated and artificially ventilated on 34% O_2_. During anesthesia the animals were placed resting on their elbows to minimize the impact on the cardiovascular system [15]. In order to avoid hypothermia, the animals were wrapped in a blanket during anesthesia

### 2.1. Membrane Fabrication

 The PCL-E membrane was fabricated by transferring a solution of 10 weight percent (wt%) PCL in CHCl_3_ to a 5 mL syringe attached to a blunt tipped 18 gauge (G) stainless steel needle. Electrospinning was then carried out through the application of a 15 kV positive voltage to the polymer solution. The solution was fed via a syringe pump at a constant mass flow rate of 1 mL/hr. Finally fibers were collected on a stainless steel grounded rotating drum until a nonwoven mat was formed.

The PCL-SNW membrane was fabricated by first preparing a polymer casting solution by dissolving PCL in dichloromethane (4 wt%) (Sigma-Aldrich). The PCL solution was then cast onto a nanoporous anodized aluminum oxide template using a spin coater (Specialty Coating Systems, Indianapolis, IN, USA). The solvent was allowed to evaporate at room temperature. Polymer melts were formed at 130°C while in contact with the nanoporous template. Nanowire length was tuned as a function of melt time. A melt time of 5 min was used to form the short nanowires of 2.5 *μ*m in length. Finally the thin-film scaffold of vertically aligned nanowires was released by etching the template in a dilute sodium hydroxide solution and allowed to air dry at room temperature.

The PCL-s membrane was fabricated on an electrochemically polished silicon wafer using a spin-cast/solvent evaporation method.

### 2.2. Surgical Procedure

 Eyes were anesthetized, dilated, disinfected, and a standard three-port core vitrectomy was performed as previously described [16]. In brief, the infusion line was secured inferiorly (Ringer Lactate; SAD, Copenhagen, Denmark), and the vitreous was removed during endoillumination using a 20 G vitrector (Karl Storz GmbH, Tuttlingen, Germany). The posterior hyaloid was meticulous removed in the visual streak and optic disc area. A subretinal bleb in the visual streak area was raised by injection of Ringer Lactate (SAD, Copenhagen, Denmark) through a 41 G cannula (ref. 1270; DORC International BV, Zuidland, the Netherlands). To gain access to the subretinal space, a retinotomy was performed in the temporal aspect of the bleb using endodiathermy (Storz Premiere, Bausch & Lomb; energy set 15%, output range 7.5 Watts nominal at 100 ohms) and automated scissors (Storz Premiere, automatic scissors).

This allowed a large piece of membrane (approx. 12 mm^2^) to be inserted in the visual streak area. DORC's combined spatula/peeling forceps was used for this (Ref. 1297, DORC, Netherlands). In order to secure the membrane, a partial fluid-air-exchange with drainage at the retinotomy site was performed after the membrane was placed subretinally. Sclera and conjunctiva were sutured with 7-0 coated vicryl (Ethicon, Norderstedt, Germany). After the procedure, intraocular pressure was evaluated with bimanual palpation, and indirect ophthalmoscopy was performed to ensure correct placement of the membrane and absence of bleeding and retinal detachment. Finally, topical application of chloramphenicol ointment was given, and the eye was patched (Kloramfenikol “DAK”; Nycomed, Roskilde, Denmark). In order to ensure reliable histology and mfERG recordings, animals with any surgical complication, such as bleeding, surgical lens damage, or retinal detachment as well as animals with significant opacities in the media were excluded from the study.

### 2.3. Follow-Up Procedure

Six weeks past surgery, animals were reanesthetized as previously described [15] with addition of a neuromuscular blocker to avoid eye movement: 2 mg/h i.v. Pancurium Bromide (Oss, Organon, Holland).

Infrared (IR) fundus imaging, mfERG (VERIS Science 5.0.1), and color fundus photos (Zeiss FF450 plus-IR) were obtained just prior to euthanasia. All color fundus photos were taken with an angle of 50 degrees. Animals were euthanized by a lethal injection of 20 mL pentobarbital 200 mg/mL (Royal Veterinary and Agricultural University, Copenhagen, Denmark) and the left membrane-transplanted eye was then enucleated. After an initial fixation for 15 min in formaldehyde 4%, the enucleated eye was divided into two parts by incision posterior to the ora serrata. Subsequently, the formaldehyde fixation was prolonged for 24 hr.

### 2.4. Histology

 The optic nerve and the visual streak were isolated from the formaldehyde fixed eyecup. The obtained specimens were paraffin embedded and sections of 4.5 *μ*m were then stained with haematoxylin and eosin (HE) and evaluated by light microscopy. The proportion of morphologically undisturbed retinal layer overlaying the transplanted membrane was measured on a micrograph. For each retinal layer, a fraction of morphologically undisturbed retina could then be calculated. This semiquantitative scoring of the histological effects on the retina enabled comparison of membranes independently of the placement of the membrane within the eye.

### 2.5. Multifocal Electroretinogram

 Multifocal ERG was recorded on both eyes as previously described [17]. In brief, eyes were dilated and a Burian Allen (VERIS Infrared Illuminating Electrode, EDI, Inc., Red Wood, CA) bipolar contact lens was placed on the cornea. Recordings were conducted using VERIS Science 5.0.1 with visual stimulus displayed on a 1.5-inch cathode ray tube monitor integrated in the stimulus camera. The left membrane-implanted eye was recorded first and always recorded within the first two hours of anesthesia. The two eyes were recorded within a timeframe of 30 min. A stimulus pattern of 241 unscaled white and black hexagons with a frame rate of 75 Hz and 16 samples per frames was used. The m-sequence exponent was 15 and the durations of recordings were 7.17 minutes. Signals were band-passes filtered outside 10–300 Hz. No spatial averaging and only first-order kernels were used.

Multifocal electroretinogram scoring of the membranes was done as previously described [17]. In brief, IR fundus photo of the left eye with the stimulus grid from the VERIS system and the corresponding Zeiss color fundus photo was aligned to identify hexagons covering membrane-supported retina. The corresponding area in the right (control) eye was identified, and the averages of the P1-amplitudes of the mfERGs derived from hexagons of the two areas were calculated and a ratio was found. ANOVA-test was used to test for equality of means between the different membrane types (SigmaPlot 11.0 for Windows, Systat Software Inc., San Jose, California, USA).

### 2.6. Brightness Analysis

 Color fundus photos were used to evaluate the brightness of the subretinally transplanted membranes. Area of interest was marked and measured in Photoshop on a scale ranging from 0 (black point) to 255 (white point) as described by Hubbard et al. [18]. The ratio between the membrane area and the optic disc brightness was used to even out differences in the fundus photo flash intensity. The brightness ratios for the three membranes were plotted using SigmaPlot.

## 3. Results

A total of 28 pigs underwent PCL-membrane implantation surgery, whereof 12 had PCL-S, 10 had PCL-E, and 6 had PCL-SNW membranes implanted.

The implanted membrane could not be found in 7 cases at followup and in 1 pig the membrane was found in the vitreous cavity. Two pigs developed intraocular bleeding that did not resolve, and in 2 pigs the retina did not reattach. One PCL-SNW had mfERG responses, but the retina was lost during histological preparation; in 4 eyes (one PCL-E, one PCL-SNW, and two PCL-S), the membrane moved so far peripherally that detection of the mfERG was not possible, and in one PCL-E the membrane had migrated intraretinally. This gives a total of 10 pigs that were included for both histological scoring and mfERG recordings (Table 1).

The different fabrication methods of PCL membranes irrefutably resulted in different degrees of flexibility. This proved to influence the ease of implantation to the eye. PCL-SNW was the easiest to maneuver to and insert in the subretinal space. The relatively stiff PCL-E gave an impression of distorting the surrounding tissue when inserted subretinally, whereas the high flexibility of the Smooth membrane made it bend and curl up during the implantation.

**Figure 1:**
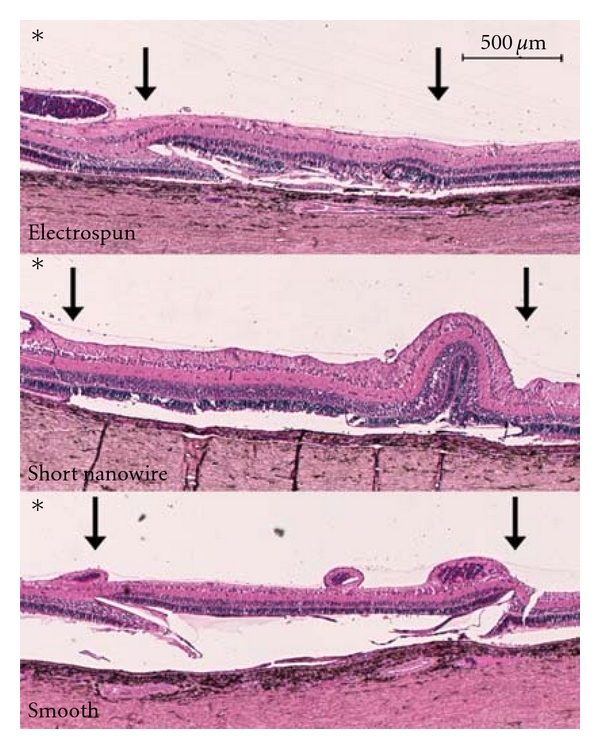
Micrograph of hematoxylin and eosin stained porcine retina after subretinally implanted poly(*ε*-caprolactone) membranes (marked with arrows). Top: Poly(*ε*-caprolactone) Electrospun PCL-E) membrane. The left edge of the membrane has penetrated up through the outer retina. Mild choroidal neovascularization is seen under the membrane and a few RPE-cells have transformed to a macrophage morphology on the outer face of the membrane. Very little of the retina from the inner nuclear layer, and outward, is preserved and no photoreceptors are preserved. Middle: Poly(*ε*-caprolactone) Short Nanowire (PCL-SNW) membrane. A retinal fold is seen over the right end of the membrane taking up approximately 30% of the length of the membrane. All retinal layers left of the fold from neurofiber to outer nuclear layer are well-preserved giving approximately 70% well-preserved retina. Half the photoreceptor outer segments are flattened but still present. Bottom: Poly(*ε*-caprolactone) Smooth (PCL-S) membrane. Both membrane edges are perforated up through the outer retina. The inner membrane-supported retinal layers are well preserved. From the inner nuclear layer, the outer retina is more disrupted with preserved morphology in only approximately half the length of the membrane. The part of fattened but still present photoreceptor outer segments is even less than the rest of the outer retina. No sign of inflammation is seen in either of the micrographs. (∗ marks the vitreous body).

Histological examination showed all the membranes to be rather well tolerated in the subretinal space with no inflammation in either retina or choroid (Figure 1). In a few cases, RPE cells on the outer face of the membranes transformed to a histiocytic morphology creating giant cells (Figure 1(top)). Choroidal neovascularization (CNV) was seen in 67% cases with PCL-E, 75% of cases transplanted with PCL-S, and in 50% of cases with PCL-SNW membranes.

It was possible to score the morphologically intact fraction of all overlying retinal layers of all included eyes. Mean values for each layer and membrane type including lowest and highest fraction are given in Figure 3. The PCL-SNW had the highest average percentage of morphologically well-preserved overlying retina for all evaluated outer retinal layers. The photoreceptor outer segment layer was disrupted over all the membranes, varying from flattened to completely gone. This layer, as the only one, is, therefore, included as preserved if present (Figure 3). All other layers varied from undisturbed to disturbed beyond recognition and only the morphologically undisturbed part of these layer were included as preserved. The inner retina was left almost intact by all membranes but one case of PCL-S (Figure 3). The one PCL-SNW that scored low on outer retinal layers did so because of a retinal fold that was scored as morphologically disturbed (Figure 1). Common for all membranes was a tendency for the edges to penetrate up through the retinal layers and in one case the membrane was found to be located completely intraretinally.

**Figure 2:**
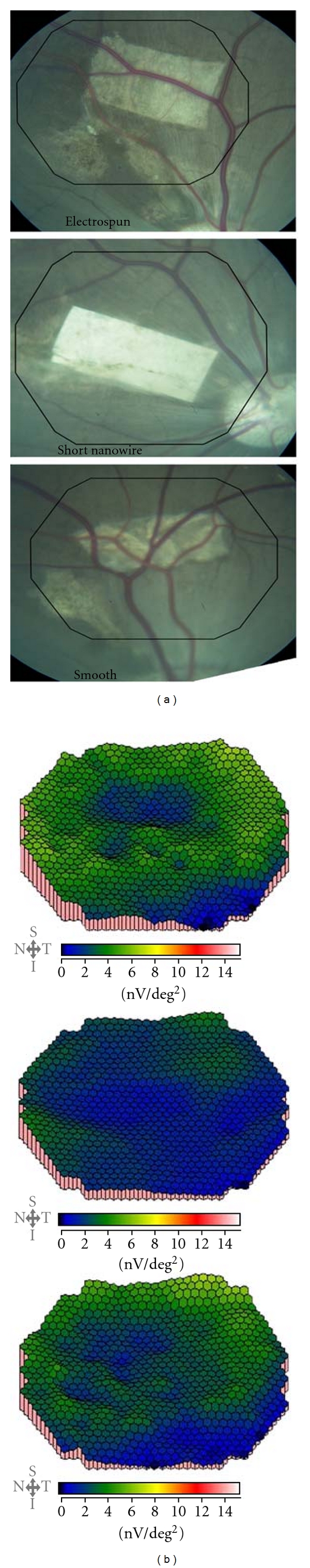
Fundus photos 6 weeks after membrane implantation (a) and corresponding 3D-presentation of amplitudes for the 241 hexagons recorded by multifocal electroretinography (b). Black circle on the fundus photo marks position of the 241-unscaled multifocal electroretinogram-grit. Color scale on the 3D-presentation ranges from 0 to 14 nV/deg^2^. Top: Poly(*ε*-caprolactone) Electrospun PCL-E) membrane. Middle: Poly(*ε*-caprolactone) Short Nanowire (PCL-SNW) membrane. Bottom: Poly(*ε*-caprolactone) Smooth (PCL-S) membrane.

 It was possible to obtain good mfERGs with acceptable signal-to-noise ratios in both the left and right eye on all included pigs. The 3D presentations of amplitudes show a general depression over the membranes when compared to adjacent retina (Figure 2). This tendency is also found for the P1-amplitude ratios (Figure 4). The depression in P1-amplitude ratio is, however, only significantly different from 1 for the PCL-S. Between the three membrane types, no significant difference in mean P1-amplitude ratios was found (Figure 4). The 3D presentation of amplitudes for the membrane adjacent retina of the three membrane types shows the interindividual difference generally seen between the pigs (Figure 2).

Fundus photos of the membranes reveal a difference in brightness of the membranes (Figure 2). This difference in brightness is significant between the PCL-SNW and the PCL-E membranes (Figure 5).

## 4. Discussion

We demonstrate, in this study, that three different modifications of poly(*ε*-caprolactone) membranes, Electrospun, Short Nanowire, and Smooth, are well tolerated in the subretinal space in pigs. None of the tested membrane variants caused an inflammatory response, but differences in the retinal damage seen could be related to the physical properties of the different membrane types.

The thickness and biocompatibility of scaffolds has previously been addressed as essential properties of tissue scaffolds for subretinal stem cell transplantation [13, 19, 20]. Firstly, the scaffold must be thin and permeable to nutrients to allow outer retinal survival. The PCL-SNW scaffolds especially fulfill this requirement, as it is one of the thinnest polymer substrates used for subretinal RPC transplantation [13]. Secondly, scaffold material must not be toxic to the surrounding tissue. PCL as a material for scaffold complies well with these requirements, as it is both highly permeable [12] and degrades in a slow manner and is less prone to leave an acidic microenvironment than polymers of higher molecular weight as the PLGA [21, 22]. Finally, to allow surgical delivery to the subretinal space, the scaffold needs to be stiff, but still flexible enough to prevent distortion of the subretinal space. The PCL-E has a very high Young's modulus (stiffness) [9], which prevented the membrane from adapting to the curvature of the eyeball, and likely caused the observed migration up into retina. In contrast, the PCL-S proved too flexible, as it tended to curl up during implantation, and therefore caused a larger surgical trauma. Over all, we find the PCL-SNW to have the best degree of flexibility and the PCL-S to have the worst.

**Figure 3:**
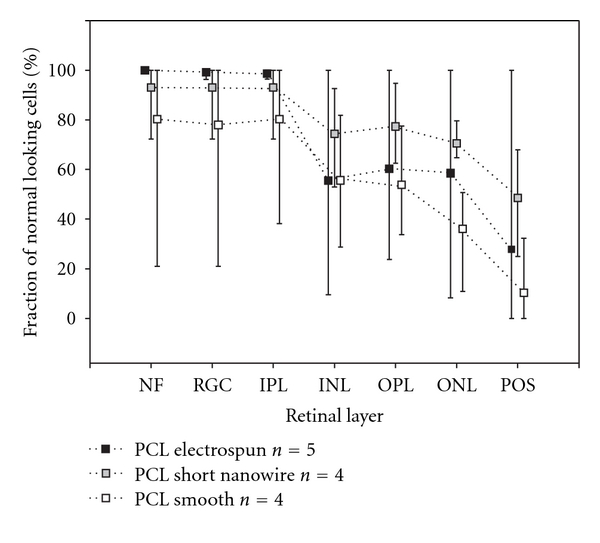
Mean fractions of morphologically intact membrane supported retinal layers for the three membrane types (squares). The lowest and highest scored fractions for each layer are given by the thin bars. Number of pigs included for histological scoring are given as *n* values in the figure.

**Figure 4:**
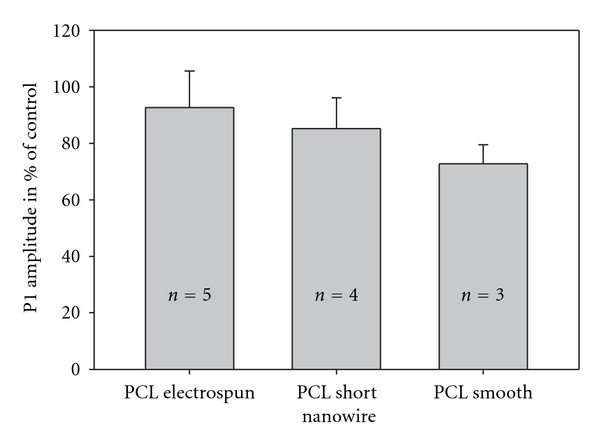
Mean P1 amplitude ratios for the three membrane-types. Ratios are given as the percentage of P1 amplitude for the membrane supported area of retina in the left eye compared to that of the corresponding area in the right untouched eye. Confidence intervals are given by the thin bars. Number of included animals for each membrane are written on the broad bars. No significant differences are seen. Only the P1 amplitude ratio of PCL Smooth is significantly different from 1 (*P* = 0.028).

**Figure 5:**
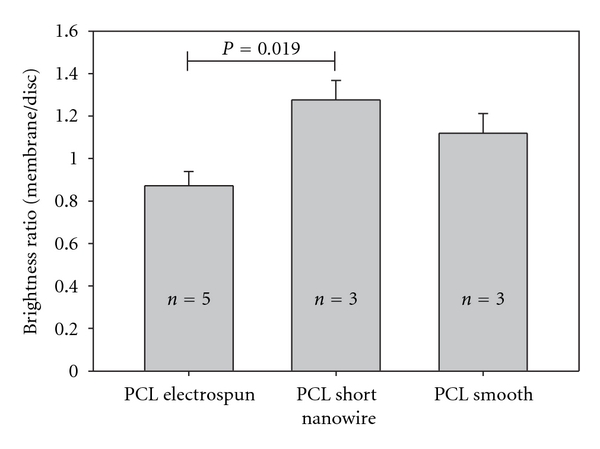
Mean brightness ratios for the three membrane types. Ratios are calculated as brightness of the membrane compared to that of the optic disc. Confidence intervals (thin bars) reveal a significant difference between the poly(*ε*-caprolactone) (PCL) Electrospun and the PCL Short Nanowire membranes. Numbers of included animals for each membrane type are written on the broad bars.

Our histological results also point towards the PCL-SNW as the best scaffold candidate of the three tested as the overlying outer retina (from inner nuclear layer and out) of the PCL-SNW had approximately 17% more preserved morphology than the PCL-E, and 25% more than the PCL-S (Figure 3). Histology showed that the inner retina was mainly left intact in all specimens, except for one smooth membrane. In one specimen with PCL-E we found intact photoreceptor layer, but the overall finding was that photoreceptors were shortened and nowhere was a completely morphologically intact layer observed. Flattening of the outer segments has previously been observed in the porcine retina after detachment but here the morphology of outer segments was restored 6 weeks after detachment and the subsequent reattachment [14]. We, therefore, regard the loss and morphological change of the outer segments to be expected and not specific to these membrane types. Besides the effect of mechanical differences between the membranes, an explanation for the different degrees of preservation could be the ability of membranes to support growing cells [13].

We believe that the tendency to curl up after implantation combined with the smooth surface is the explanation for the high number of PCL-S membranes not found subretinally at followup (Table 1). As the PLC degrades very slowly and is found so well preserved in the other eyes, we do not believe that the membranes not found after 6 weeks had dissolved. They either migrated subretinally so far anteriorly, that they were not visible on neither fundus photo nor histology or, more likely, they were displaced through the retinotomy, as the one seen at the posterior lens capsule, and were, therefore, not seen during the preparation process.

Choroidal neovasculariztion often occurs with damage to Bruch's membrane [23] and was in this study found in more than half the included eyes. We do not expect the presence if the electrically inactive CNV to influence the mfERG directly. Indirectly, however, CNV could influence the mfERG via scatter. Again we do not expect this to affect the mfERG of the membrane supported retina as the CNV is located under the implanted membranes.

The intentional use of mfERG as an in vivo measurement of cell survival is compromised in this study. If mfERG is to be used for assessment of survival of transplanted cells, a significant depression in mfERG amplitude ratio in the control situation (the naked membrane) is mandatory. On the 3D presentation of the mfERG, there was a tendency for depression of the mfERG signals over the membranes, but when compared to the control eye only PCL smooth showed a significant decrease in P1 amplitude. Although histology revealed marked differences in the destruction of outer retinal layers covering the different membrane types, we could not show any significant differences in P1 amplitude ratios. This was further complicated by the observation of significantly higher brightness ratio for PCL-SNW compared to PCL-E and so a correlation between brightness ratio and mfERG P1 amplitude ratios could not be demonstrated, as was previously shown for PLGA membranes [17]. We explain this dissimilarity by the presence of residual functional photoreceptors overlaying the bright membrane, resulting in mfERG amplitudes composed of both signals from functional local retina and from light scatter in various degrees.

## 5. Conclusion

In conclusion, PCL derivatives are well tolerated in the subretinal space in pigs. Among the three PCL scaffolds tested, PCL short nanowire seems to be the best candidate for future use as a scaffold in RPC transplantation.

## Figures and Tables

**Table 1:** Number of implanted, included, and excluded polymer scaffolds, by type.

	No. of eyes (total)	No. of eyes (histology)	No. of eyes (mfERG)	No. of eyes (mfERG and histology)	Exclusions
PCL Electrospun	10	6	5	4*	1 bleeding; 2 retinal detachments; 1 membrane not found at follow up

PCL Short Nanowire	6	4	4	3	1 membrane not found at follow up

PCL Smooth	12	5	3	3	1 bleeding; 1 membrane dislocated to corpus vitreum; 5 membranes not found at follow up

*One membrane migrated intraretinally and was not scored histologically.

## Abbreviations

RPC: Retinal progenitor cell
PCL: Poly(*ε*-caprolactone)
PCL-E: Poly(*ε*-caprolactone) electrospun,
PCL-SNW: Poly(*ε*-caprolactone) short nanowire
PCL-S: Poly(*ε*-caprolactone) smooth
mfERG: Multifocal electroretinogram
i.v.: Intravenous infusion
G: Gauge
IR: Infrared
HE: Haematoxylin and eosin
CNV: Choroidal neovascularization.
